# Examining the impact of excessive daytime sleepiness on utility scores in patients with obstructive sleep apnoea and/or narcolepsy in five European countries

**DOI:** 10.1186/s12883-022-02827-7

**Published:** 2022-08-25

**Authors:** M. Janelle Cambron-Mellott, Sam Mettam, Vicky W. Li, John C. Rowland, JeanPierre Coaquira Castro

**Affiliations:** 1 Cerner Enviza, Malvern, PA USA; 2 Jazz Pharmaceuticals, Oxford, UK; 3 Formerly Jazz Pharmaceuticals, Palo Alto, CA USA

**Keywords:** Health states utilities, Excessive daytime sleepiness, Obstructive sleep apnoea, Narcolepsy, Epworth Sleepiness Scale

## Abstract

**Background:**

Excessive daytime sleepiness (EDS) is a cardinal symptom of narcolepsy and affects many patients with obstructive sleep apnoea (OSA). EDS is associated with reduced quality of life, increased accident risk, and poor workplace performance. Given the impact of EDS, the ability to predict health-related utility from sleepiness is valuable for examining the cost effectiveness of novel treatments. The aim of this study was to examine the association between EDS and EQ-5D in patients with OSA and/or narcolepsy by modelling EQ-5D utility scores from Epworth Sleepiness Scale (ESS) scores.

**Methods:**

Data were obtained from the Europe 2016/2017 National Health and Wellness Survey, an online, general population survey, designed to represent the age and gender composition of each country’s adult population. Analyses included 2,348 patients self-reporting symptomatic and diagnosed OSA (*n* = 2,277), narcolepsy (*n* = 48), or both (*n* = 23). Multivariable models were used to examine ESS as a predictor of EQ-5D utility while adjusting for covariates of interest. Results were validated following the National Institute for Health and Care Excellence Decision Support Unit guidelines for predictive modelling.

**Results:**

Utility decreased as EDS severity increased (no EDS: 0.711 ± 0.251, mild: 0.685 ± 0.261, moderate: 0.643 ± 0.268, severe: 0.559 ± 0.323). Whereas participants with only OSA or only narcolepsy did not differ in utility, those with both conditions had lower scores (0.685 ± 0.266 and 0.627 ± 0.325 vs. 0.439 ± 0.340, respectively). Piecewise linear regression identified a single breakpoint at ESS score of 11.29. In the final model, for each point increase in ESS score, the corresponding decrease in EQ-5D utility was larger among patients with ESS scores ≥ 12 compared to patients with ESS scores ≤ 11 (model slopes: -0.0131 vs. -0.0026, respectively). Findings from the validation sample confirmed these results.

**Conclusions:**

This study demonstrates the impact of sleepiness on quality of life (QoL) and its negative impact irrespective of sleep condition (OSA or narcolepsy). The breakpoint identified is relatively consistent with the established ESS cutoff score ≥ 11, which demarcates pathological sleepiness. Furthermore, as EDS severity worsens (increases) on the ESS, the impact on QoL is greater.

**Supplementary Information:**

The online version contains supplementary material available at 10.1186/s12883-022-02827-7.

## Background

Excessive daytime sleepiness (EDS) is the primary concern for many patients with sleep disorders and is characterized by the inability to stay awake, alert, and optimally functional throughout the day [1]. EDS has been estimated to affect between 2.5% and 26.1% of the general population, varying depending on the definition applied and method of assessment [2].

The most common causes of EDS are insufficient sleep and poor sleep quality [3]. Sleep continuity is a crucial factor in determining sleep quality, with sleep breathing disorders such as sleep apnoea representing a common cause of sleep disruption. Many neurological and psychiatric disorders are associated with EDS [3, 4].

In narcolepsy, a chronic disorder characterized by the dysregulation of the sleep/wake cycle, EDS is the cardinal symptom [5, 6] and is generally present in all patients [5–8]. Narcolepsy is characterized by recurrent, uncontrollable brief episodes of sleep and lapses in consciousness; other symptoms of narcolepsy include hypnagogic and hypnopompic hallucinations, cataplexy, sleep paralysis, disrupted nocturnal sleep, and, in some cases, automatic behaviours (i.e. abnormal waking activities) [3, 9]. The prevalence of narcolepsy in the general population is relatively low, estimated to be around 0.04% [2].

EDS is also a prominent symptom in obstructive sleep apnoea (OSA), which is characterized by frequent partial arousals that occur throughout sleep as a result of recurrent episodes of partial or complete collapse of the upper airway [10]. OSA has a high prevalence in the general population; a 2019 study estimates OSA affects 936 million adults aged 30 to 69 years globally, or approximately 12% of the world’s population [11]. This study extrapolated country-specific prevalence rates, including five European countries (France, Germany, Italy, Spain, United Kingdom [UK]), with prevalence of moderate to severe OSA estimated at 21.4%, ranging from 4.8% in the UK to 36.3% in France [11]. While many studies examining the prevalence of OSA in Europe have shown varying rates between countries, one consistent finding is that the prevalence of OSA is higher in men than women [12–17]. The prevalence of OSA associated with EDS is approximately 3 to 7% for men and 2 to 5% for women in the general population [10], and it has been found that residual EDS remains in up to 13% of patients even after continuous positive airway pressure (CPAP) treatment [18].

EDS is a significant public health problem with serious economic consequences. EDS is associated with poor health-related quality of life (HRQoL), poor performance in the workplace and increased risk of accidents [19]. Narcolepsy is associated with significant impact on HRQoL [20], with EDS emerging as a strong predictor of poor HRQoL in patients with narcolepsy [21–24]. Furthermore, treatment with stimulants or wakefulness-promoting agents at dosages high enough to alleviate EDS in patients with narcolepsy often leads to adverse effects, although more recently developed wakefulness-promoting agents have better risk:benefit profiles than traditional stimulants [3, 25, 26].

There is also increasing evidence that higher levels of EDS in patients with OSA account for an increased burden of disease. This includes an increased risk of cardiovascular disease [27], depression [28], and diabetes [29], greater deficits in work productivity [30], increased health-care utilization [31], and worse HRQoL [32, 33].

EDS may be assessed by the Epworth Sleepiness Scale (ESS), an 8-item patient-reported outcome scale that scores respondents on how likely they are to doze off during certain daily activities (e.g., sitting and reading, watching TV, etc.) [34]. Given the impact of EDS, the ability to predict health-related utility from ESS scores is valuable for examining the cost effectiveness of treatments for EDS. Indeed, while developing the technology appraisal guidance for CPAP machines, the National Institute for Health and Clinical Excellence (NICE; the UK’s health technology assessment agency) assessment group identified three studies that examined ESS score and SF-36 and/or EQ-5D data, and using this data, used a linear regression model to predict utility from ESS scores [35]. This led to the development of a mapping algorithm to convert ESS scores into utility scores in the 2008 NICE CPAP appraisal; the cost effectiveness of CPAP machines was then examined by applying the mapping algorithm to data on mean difference in ESS scores between individuals treated with CPAP machines compared to those treated with placebo or compared to those treated with dental devices [35]. However, the mapping algorithm based on EQ-5D was generated based on data from only 94 patients with OSA [36].

The current study aimed to expand on the previous research undertaken by NICE by including data from a larger number of patients, including patients with OSA and/or narcolepsy, and examining alternative models to simple linear regression to explain the association between EDS and EQ-5D utility scores. Therefore, the objective of the current study was to examine the association between EDS and HRQoL in patients with OSA and/or narcolepsy to predict EQ-5D utility scores from ESS scores.

## Methods

### Data source and procedures

This retrospective, cross-sectional study used data from 5 European countries (France, Germany, the UK, Italy, and Spain) from the 2016 and 2017 National Health and Wellness Survey (NHWS), a self-administered, internet-based questionnaire completed by adults (aged 18 years or older). The NHWS is designed to reflect the general population of each country surveyed using quota sampling based on age and sex for each country.

Potential respondents were identified primarily through participation in opt-in online survey panels. In 2016, online panel recruitment in Germany and Italy was supplemented by computer-assisted web interviews, in which respondents 65 years of age or older were recruited on the telephone and have the choice to complete the interview by phone. This was done to further ensure representativeness, particularly in the population 65 + years old.

Respondents were included if they self-reported having been clinically diagnosed with OSA and/or narcolepsy, self-reported experiencing OSA and/or narcolepsy in the past 12 months, and completed the ESS. Importantly, although the NHWS does not ask patients to specify their type of sleep apnoea (i.e. obstructive, central), this study made the analytic decision to accept *sleep apnoea* as adequately capturing and representing OSA based on the significantly higher prevalence of OSA compared to central sleep apnoea, with recent analysis of baseline data from a large community-based cohort study (i.e. the Sleep Heart Health Study) calculating a prevalence of 47.6% and 0.9%, respectively, among adults aged 40 years and over [37]. This same approach was used by Stepnowsky et al. [38].

In 2017, all respondents who reported experiencing OSA and/or narcolepsy in the past 12 months completed the ESS module, whereas in 2016 only a random subset of patients eligible to answer module questions did so. In cases where respondents participated in multiple years, the most recent data were analysed.

The protocol and questionnaire for the 2016 and 2017 NHWS were reviewed and granted exemption by the Pearl Institutional Review Board (IRB) as it was determined this study met the exemption requirements under 45CFR46.101(b)(2). All respondents provided informed consent.

### Measures

#### Predictor variable

For quantification of EDS status, the predictor variable for this study was the ESS score, ranging from 0–24 (higher scores indicating worse daytime sleepiness).

The ESS was analysed as a continuous score and as a categorical measure of EDS status, using two cutoffs sets. The first set used the following thresholds to delineate ESS scores: ≤ 10 ‘no EDS’, 11–12 ‘mild EDS’, 13–15 ‘moderate EDS’ and > 16 ‘severe EDS’. The threshold delineating non-pathological from pathological levels of sleepiness (≥ 11) was developed in an Australian population [39], and the thresholds delineating the degree of EDS were empirically developed based on data reported by patients with narcolepsy [40]. These cutoffs are widely used in the United States (US) and abroad (except in the UK) and will be referred to in this study as US/Rest of World (RoW) cutoffs [40, 41]. The second set utilized thresholds found on the NICE Clinical Knowledge Summaries site [42]. They are referred to here as UK cutoffs with an ESS score of ≤ 10 indicating ‘no EDS’, 11–14 ‘mild EDS’, 15–18 ‘moderate EDS’, and 19–24 ‘severe EDS’.

#### Covariates and other variables of interest

Respondents were categorized according to their sleep disorder: ‘OSA without narcolepsy’, ‘narcolepsy without OSA’, or both ‘OSA and narcolepsy’. Additionally, information was collected on a variety of sociodemographic and health characteristics including age, sex, country, marital status, education, labour force participation, employment status, annual household income, body mass index (BMI) category, smoking status, alcohol use, exercise activity, and Charlson Comorbidity Index (CCI) score.

#### Outcome variable

The outcome variable for this study was the EQ-5D utility score. The EQ-5D utility score is derived from responses to the EQ-5D-5L, a widely-used, generic survey instrument which measures health status and consists of five dimensions: mobility, self-care, usual activities, pain/discomfort, and anxiety/depression; with 5-point rating scales for each dimension [43].

Each health state can be assigned a summary utility score based on representative preference weights for the health state. Health state utility scores generally range from less than 0 (where 0 is the score of a health state equivalent to dead; negative values representing health states worse than dead) to 1 (the score equivalent to full health), with higher scores indicating higher utility [23]. These utility scores were calculated by mapping the five-level descriptive system onto the three-level valuation set using the mapping function developed by van Hout et al [44]. Health states were mapped using their own country-specific value set.

### Statistical analysis

Descriptive analyses, including means and standard deviations (SD) for continuous variables and counts and percentages for categorical variables, were conducted to examine demographic and health variables by OSA/narcolepsy status. Descriptive statistics, using means and SDs, were also used to examine EQ-5D utility scores by OSA/narcolepsy status and by EDS status using the US/RoW cutoffs.

Multivariable models were used to develop an equation to predict EQ-5D utility scores from ESS scores, while adjusting for variables of interest. The included covariates were OSA/narcolepsy status, age, CCI, sex, marital status, income, BMI, smoking status, alcohol use, and exercise.

Four models were initially run; three models utilized generalized linear models (GLMs), specifying normal distribution and identify link function, to examine EQ-5D utility scores by continuous ESS scores (model *(a)*), and categorical EDS status (no EDS [reference], mild EDS, moderate EDS, and severe EDS) using US/RoW cutoffs (model *(b)*) and using UK cutoffs (model *(c)*). The fourth model utilized piecewise linear regression to identify whether there was a breakpoint in the linear relationship between ESS scores and EQ-5D utility scores (model *(d)*).

After reviewing results from these four models, a linear spline regression (model *(e)*) was run to improve interpretability. Specifically, the explanatory variable was portioned into intervals reflecting the segments identified in *(d)*, utilizing discrete ESS score cutoffs, and a separate line segment was fit to each interval. An interaction term was included in the final model to examine whether ESS scores and OSA/narcolepsy status interacted to predict EQ-5D utility scores.

Model fit was assessed in terms of deviance, Akaike information criterion (AIC), and Bayesian information criterion (BIC). *P*-values were reported for continuous and categorical ESS scores, depending on the model. Due to no adjustments for multiplicity, the *P*-values presented are nominal.

The final model equation was validated following the NICE Decision Support Unit guidelines for predictive modelling [35]. This included splitting the full sample into estimation and validation samples using a 70/30 split for additional analyses, running bivariate comparisons between the estimation and validation samples across all covariates/outcomes in the model, running the final model on the estimation and validation samples and assessing model fit through root mean square error (RMSE), assessing bias in prediction by calculating RMSE by quartiles of EQ-5D and plotting observed vs. predicted values, and running a piecewise linear regression on the estimation sample to determine if a breakpoint in ESS score existed and then compare to the breakpoint identified in the full sample.

## Results

A total of 2,348 respondents self-reported both symptomatic (experienced in the last 12 months) and physician-diagnosed ‘OSA without narcolepsy’ (*n* = 2,277), ‘narcolepsy without OSA’ (*n* = 48), or both ‘OSA and narcolepsy’ (*n* = 23). Participants with OSA without narcolepsy were on average older (mean ± SD: 59.3 ± 12.5 years) than participants with OSA and narcolepsy (53.3 ± 14.1 years) and participants with narcolepsy without OSA (49.0 ± 17.8 years). The majority of participants in both OSA groups were male (70.5% of ‘OSA without narcolepsy’ participants and 69.6% of ‘OSA and narcolepsy’ participants), while just under half of the ‘narcolepsy without OSA’ participants were male (47.9%). Participants with narcolepsy reported higher proportions of severe EDS (35.4% of participants with narcolepsy without OSA and 39.1% of participants with OSA and narcolepsy) than participants with OSA without narcolepsy (11.9%). In addition, participants with narcolepsy had greater comorbidity burden (mean CCI, 1.3) than participants with OSA (mean CCI, 0.6). Other participant characteristics are presented in Table 1.

**Table 1:** Participant characteristics by OSA/narcolepsy status

	**OSA without narcolepsy**	**Narcolepsy without OSA**	**OSA and narcolepsy**
**Characteristic**	**(*N* = 2,277)**	**(*N* = 48)**	**(*N* = 23)**
Age, years, mean (SD)	59.3 (12.5)	49.0 (17.8)	53.3 (14.1)
Male, n (%)	1,606 (70.5)	23 (47.9)	16 (69.6)
Country, n (%)			
France	707 (31.0)	16 (33.3)	6 (26.1)
Germany	689 (30.3)	13 (27.1)	6 (26.1)
UK	334 (14.7)	7 (14.6)	5 (21.7)
Italy	236 (10.4)	7 (14.6)	5 (21.7)
Spain	311 (13.7)	5 (10.4)	1 (4.3)
Married/living with partner, n (%)	1,591 (69.9)	24 (50.0)	12 (52.2)
University degree, n (%)	804 (35.3)	20 (41.7)	8 (34.8)
Annual household income, n (%)			
Low (< €/£20,000)	587 (25.8)	17 (35.4)	8 (34.8)
Medium (€/£20,000 to €/£39,999)	903 (39.7)	22 (45.8)	8 (34.8)
High (€/£40,000 or more)	622 (27.3)	7 (14.6)	6 (26.1)
CCI mean (SD)	0.6 (1.2)	1.3 (2.9)	2.0 (2.6)
Overweight/obese (BMI, ≥ 25 kg/m^2^), n (%)	1,870 (82.1)	23 (47.9)	17 (73.9)
Smoking status, n (%)			
Never smoker	711 (31.2)	12 (25.0)	4 (17.4)
Former smoker	1,032 (45.3)	20 (41.7)	10 (43.5)
Current smoker	534 (23.5)	16 (33.3)	9 (39.1)
Alcohol use, yes, n (%)	1,747 (76.7)	36 (75.0)	15 (65.2)
Exercised ≥ 1 time in past month, n (%)	1,147 (50.4)	24 (50.0)	11 (47.8)
EDS status, n (%)			
No EDS (ESS, 0–10)	1,530 (67.2)	18 (37.5)	9 (39.1)
Mild EDS (ESS, 11–12)	221 (9.7)	7 (14.6)	2 (8.7)
Moderate EDS (ESS, 13–15)	256 (11.2)	6 (12.5)	3 (13.0)
Severe EDS (ESS, 16–24)	270 (11.9)	17 (35.4)	9 (39.1)

*BMI* Body mass index, *CCI* Charlson Comorbidity Index, *EDS* Excessive daytime sleepiness, *ESS* Epworth Sleepiness Scale, *N* Number of participants, *n* number of participants with observations, *OSA* Obstructive sleep apnoea, *SD* Standard deviation

Participants with only OSA or only narcolepsy did not differ in EQ-5D utility scores, but those with both conditions had lower scores (0.685 ± 0.266 vs. 0.627 ± 0.325 vs. 0.439 ± 0.340, respectively). When examining EQ-5D utility scores by EDS status, utility decreased as EDS severity increased, from 0.711 ± 0.251 for participants with no EDS to 0.559 ± 0.323 for participants with severe EDS (Table 2).

**Table 2:** EQ-5D utility scores by EDS status

	**No EDS (ESS, 0–10)**	**Mild EDS (ESS, 11–12)**	**Moderate EDS (ESS, 13–15)**	**Severe EDS (ESS, 16–24)**
	**(*N* = 1,557)**	**(*N* = 230)**	**(*N* = 265)**	**(*N* = 296)**
EQ-5D utility scores, mean (SD)	0.711 (0.251)	0.685 (0.261)	0.643 (0.268)	0.559 (0.323)

*EDS* Excessive daytime sleepiness, *ESS* Epworth Sleepiness Scale, *N* Number of participants, *SD* Standard deviation

Models were run in accordance to the steps outlined in the methods. Parameter estimates for the ESS scores of all models are presented in Table 3. Parameter estimates for all variables are available in Supplementary Table S1 (see Additional file 1). Model fit is presented in Table 4.

**Table 3:** Parameter estimates for the ESS scores of the models predicting EQ-5D utility scores

Model	Estimate	SE	*P*
*(a)* GLM—ESS score as a continuous variable	-0.0068	0.0009	< 0.001
*(b)* GLM—US/RoW cutoffs (reference 0–10)			
Mild EDS (ESS, 11–12)	-0.0134	0.0174	0.44
Moderate EDS (ESS, 13–15)	-0.0505	0.0165	0.002
Severe EDS (ESS, 16–24)	-0.1132	0.0159	< 0.001
*(c)* GLM—UK cutoffs (reference 0–10)			
Mild EDS (ESS, 11–14)	-0.0268	0.0138	0.05
Moderate EDS (ESS, 15–18)	-0.0789	0.0167	< 0.001
Severe EDS (ESS, 19–24)	-0.1513	0.0236	< 0.001
*(d)* Piecewise linear with breakpoint at 11.29			
ESS Slope 1 (ESS, < 11.29)	-0.0028	0.0018	0.13
ESS Slope 2 (ESS, > 11.29)	-0.0134	0.0031	< 0.001
*(e)* Linear spline with breakpoint at 11			
ESS, 0–11	-0.0026	0.0016	0.11
ESS, 12–24	-0.0131	0.0022	< 0.001

Note: *P*-values for models a-c were calculated using Wald chi-square tests. *P*-values for models d-e were calculated using t-tests. Due to no adjustments for multiplicity, *P*-values presented are nominal

*EDS* Excessive daytime sleepiness, *ESS* Epworth Sleepiness Scale, *GLM* Generalized linear model, *RoW* Rest of world, *SE* Standard error, *UK* United Kingdom, *US* United States

**Table 4:** Fit indices for models run

Model	Deviance	df	Deviance/df	AIC	BIC
*(a)* GLM—ESS continuous	142.33	2333	0.061	113.497	205.678
*(b)* GLM—US/RoW cutoffs: No EDS (ESS, 0–10), mild EDS (ESS, 11–12), moderate EDS (ESS, 13–15), severe EDS (ESS, 16–24)	141.91	2325	0.061	122.601	260.872
*(c)* GLM—UK cutoffs: No EDS (ESS, 0–10), mild EDS (ESS, 11–14), moderate EDS (ESS, 15–18), severe EDS (ESS, 19–24)	141.99	2331	0.061	111.912	215.615
*(d)* Piecewise linear: ESS score 0–11, ESS score 12–24	141.74	2331	0.061	107.752	211.456
*(e)* Linear spline with breakpoint at 11: ESS score 0–11, ESS score 12–24	141.74	2332	0.061	105.809	203.751

*AIC* Akaike information criterion, *BIC* Bayesian information criterion, *df* degrees of freedom, *EDS* Excessive daytime sleepiness, *ESS* Epworth Sleepiness Scale, *GLM* Generalized linear model, *RoW* Rest of world, *UK* United Kingdom, *US* United States

The piecewise linear regression (model *(d)*) identified a single breakpoint at the ESS score of 11.29 with a change of -0.0107 in slope (*P* = 0.003). As this model identified where breakpoints existed in the data and had the lowest deviance, AIC, and second-lowest BIC (Table 4), the breakpoint identified in this model informed the choice of the final model. As the ESS yields discrete scores, rather than estimating slopes based on the breakpoint of 11.29, a linear spline regression model with a breakpoint at ESS score of 11 (the closest applicable value of ESS to 11.29) was used to develop the final model *(e)*. The slope for segment 0–11 was -0.0026 and the slope for segment 12–24 was -0.0131. This model showed best fit to the data (Table 4).

Based on the final model, the equation for estimating EQ-5D utility scores using parameter estimates was found to be:

$$\mathrm{EQ}-5\mathrm{D utility score }= 0.6080 - 0.0026*[\mathrm{ESS Score }0-11] - 0.0131*[\mathrm{ESS Score }12-24] - 0.0260*[\mathrm{OSA without narcolepsy}] - 0.1622*[\mathrm{OSA and narcolepsy}] + 0.0006*[\mathrm{Age}] - 0.0352*[\mathrm{CCI}] - 0.0579*[\mathrm{Female}] + 0.0295*[\mathrm{Married}] + 0.0496*[\mathrm{Medium Income}] + 0.0534*[\mathrm{High Income}] - 0.0095*[\mathrm{BMI above }25] + 0.0045*[\mathrm{Former Smoker}] - 0.0028*[\mathrm{Current Smoker}] + 0.0496*[\mathrm{Drinks alcohol}] + 0.1060*[\mathrm{Exercises}]$$

Each point of increase in ESS scores between 0 to 11 is associated with a 0.0026 point of decrease in EQ-5D scores, whereas each point of increase in scores between 12 to 24 is associated with a 0.0131 point of decrease in EQ-5D scores. The final model was run with the interaction of sleep disorder status with ESS scores. Although an interaction occurred between the ‘OSA and narcolepsy’ group and ESS scores of 0–11 (*Β* = 0.0666, *P* = 0.006), the final model excluded this interaction term due to its small sample size, as well as the complexity of the interaction, which did not significantly add to the usefulness of the model.

### Results of validation and estimation samples

The results from the validation sample supported the findings of the final linear spline regression model and are presented in Supplementary Tables S2, S3 and S4 and Supplementary Figures S1 and S2 (see Additional file 1). The values of the outcome, predictor, and covariates were similar between the estimation and validation samples (data not shown), as were the parameter estimates between the full and estimation samples (Table S2, see Additional file 1).

Due to the limited range of predicted EQ-5D index scores, the model is shown to be less predictive at the tails of the observed EQ-5D index distribution (i.e. 1st and 4th quartiles of EQ-5D, as opposed to the 2nd and 3rd quartiles), resulting in the model underpredicting for those with the lowest and highest utilities, explaining about 18% of the variance in EQ-5D overall (Table S3, see Additional file 1).

Finally, results of the piecewise linear regression model *(d)* on the estimation sample identified a single breakpoint at ESS score 11.00 (Table S4, see Additional File 1).

## Discussion

EDS manifests enormous costs in terms of health, economic and societal impact [19]. Quantifying the effect of EDS on HRQoL is crucial for assessing effective treatments, comparing treatment outcomes in clinical practice, and determining the cost effectiveness of various treatment modalities. While the ESS is one of the most commonly used subjective measures of sleep propensity in research and clinical settings, it does not provide the health-state utility measures necessary for cost-effectiveness models.

To our knowledge, this is the largest sample used to predict utility scores from ESS scores in patients with OSA and/or narcolepsy. This study successfully expands on the previous research and presents an equation for estimating EQ-5D utility scores using a linear spline regression model with a single breakpoint, based on data from 2,348 respondents that are representative of the country-specific age and sex compositions of the included countries. The relationship established in this study provides a necessary crosswalk for when ESS scores are solely available.

A single breakpoint was identified between 11 and 12 on the ESS, indicating that the slope of the regression model changes between ESS scores ≤ 11 and ≥ 12 (-0.0026 vs. -0.0131, respectively). This suggests that patients within these two segments may be differently impacted by changes in their sleepiness. Specifically, for each point increase in the ESS score, the corresponding decrease in the EQ-5D utility score was larger among those with ESS scores of ≥ 12, than for the group having ESS scores ≤ 11. Interestingly, the breakpoint of the final model nearly aligns with the standard and widely-accepted cutoff on the ESS, where ≤ 10 corresponds to non-pathological levels of daytime sleepiness and ≥ 11 to EDS [39].

Even though data is limited due to the small number of patients with narcolepsy included in this study, utility scores did not differ by disease status (i.e. OSA or narcolepsy) but collectively decreased as EDS severity increased. This suggests that sleepiness impacts utility scores, regardless of a patient’s disease status. This is consistent with previous research on various health and social outcomes that show the strong effect of sleepiness irrespective of disease status [27–33].

The strength of this study is that the model is based on data from a large number of respondents with OSA and/or narcolepsy, including a sample almost 25 times greater than the previous analytic population used to study the association between ESS scores and EQ-5D utility scores [35]. Despite the large sample in this study, only a small number of respondents with narcolepsy were included. It should be noted that the population-based nature of the NHWS survey provided epidemiologically consistent patients counts, as the literature has documented the lower prevalence of narcolepsy compared to OSA [2, 11].

There is a potential for selection bias in this sample, as the online nature of the NHWS meant that individuals with limited or lack of internet access were less likely to participate, which could limit generalizability, especially with regards to the elderly population. However, the NHWS panel administrators attempted to have the panel mirror the population as closely as possible by controlling for age and gender during sampling. Furthermore, self-reported data is subject to recall bias, self-presentation bias and respondent fatigue. However, self-presentation and recall bias were kept to a minimum by using validated self-reported survey measures designed to eliminate these issues (e.g., the EQ-5D is based on respondents’ health ‘today’). Respondent fatigue was kept to a minimum by limiting NHWS respondents to taking no more than 12 surveys per year.

Another limitation of self-reported data is that the respondent-entered data cannot be validated. Nevertheless, the self-reported nature of the data utilized in this study possesses the advantage of being both uniformly collected across the five European countries, as well as intentionally representative of the age and gender composition of the general adult population in each country.

Given the cross-sectional nature of the data, statements of causality cannot be made from the study results. Another limitation is that measured variables were accounted for in the regression, yet there is the possibility of groups differing on unmeasured variables that may have an impact on outcomes. One such unmeasured variable is current use of pharmacotherapy prescribed for the treatment of EDS, which was not systematically captured in the survey. Future research could look to address this through a more tailored survey that captures potentially significant variables in order to further understand the association between ESS scores and EQ-5D utility scores.

Finally, we did not examine an alternative linear spline regression model using segments that correspond to the established intervals of non-pathological levels of EDS (ESS score 0–10) and pathological EDS (ESS score 11–24), despite the proximity of our final model results to this breakpoint. While it is possible that such a model would indicate adequate fit, the segments used to develop the final equation were based on where the best breakpoint(s) existed for the given data and were confirmed with the validation analyses.

## Conclusions

The results from this large, representative, population-based study across 5 European countries provide insight into the impact of sleepiness on HRQoL. Specifically, EQ-5D utility scores decrease more significantly among those with EDS relative to those with non-pathological levels of sleepiness. These results emphasize the importance of treating and effectively managing patients’ EDS associated with OSA and/or narcolepsy, as a return to a non-pathological level of sleepiness through successful treatment has the potential for significant improvements in QoL. Specifically, these findings reinforce the current notion that treatment for EDS should be targeted in patients with ESS scores ≥ 11 (the identified breakpoint in the current study), as scores within this range indicate impaired health status, poor quality of life, and increased socioeconomic burden.

## Supplementary Information

**Additional file 1:** **Table S1.** Parameter estimates for the models run. **Table S2.** Parameter estimates for linear spline regression model: estimation sample. **Table S3.** Fit statistics of estimation sample: overall and by EQ-5D quartile. **Table S4.** Slope estimates for piecewise linear regression model with breakpoint of ESS score 11.00: estimation sample. **Figure S1.** Observed versus predicted EQ-5D utility: estimation sample EQ-5D: EuroQoL- 5 Dimensions. **Figure S2.** Residual plot for observed versus predicated EQ-5D utility: estimation sample EQ-5D: EuroQoL- 5 Dimensions

## Data Availability

The data that support the findings of this study are available from Cerner Enviza but restrictions apply to the availability of these data, which were used under license for the current study, and so are not publicly available. Data can be made available for non-commercial use from the authors upon reasonable request and with permission of Cerner Enviza.

## Abbreviations

AIC: Akaike Information Criterion
BIC: Bayesian Information Criterion
BMI: Body Mass Index
CCI: Charlson Comorbidity Index
CPAP: Continuous Positive Airway Pressure
EDS: Excessive Daytime Sleepiness
ESS: Epworth Sleepiness Scale
GLM: Generalized Linear Model
HRQoL: Health-related Quality of Life
IRB: Institutional Review Board
NHWS: National Health and Wellness Survey
NICE: National Institute for Health and Clinical Excellence
OSA: Obstructive Sleep Apnoea
QoL: Quality of Life
RMSE: Root Mean Square Error
RoW: Rest of World
SD: Standard Deviation
UK: United Kingdom
US: United States
