# Supplementary material for: Examining the impact of excessive daytime sleepiness on utility scores in patients with obstructive sleep apnoea and/or narcolepsy in five European countries
Source: BMC Neurol. 2022 Aug 25;22:317. doi: 10.1186/s12883-022-02827-7 (PMC9404621; doi:10.1186/s12883-022-02827-7)
Supplement: Supplementary file 1 — Additional file 1: Table S1. Parameter estimates for the models run. Table S2. Parameter estimates for linear spline regression model: estimation sample. Table S3. Fit statistics of estimation sample: overall and by EQ-5D quartile. Table S4. Slope estimates for piecewise linear regression model with breakpoint of ESS score 11.00: estimation sample. Figure S1. Observed versus predicted EQ-5D utility: estimation sample EQ-5D: EuroQoL- 5 Dimensions. Figure S2. Residual plot for observed versus predicated EQ-5D utility: estimation sample EQ-5D: EuroQoL- 5 Dimensions [file 12883_2022_2827_MOESM1_ESM.pdf]

Additional File 1

Table S1. Parameter estimates for the models run

Model	Estimate	SE
(a) GLM - ESS score as a continuous variable		
(Intercept)	0.6241	0.0475
ESS Score	-0.0068	0.0009
OSA without narcolepsy	-0.0221	0.0367
OSA and narcolepsy	-0.1641	0.0627
Age	0.0006	0.0004
CCI	-0.0353	0.0041
Female	-0.0582	0.0119
Married	0.0287	0.0118
Medium Income (20,000-39,999)	0.0494	0.0124
High Income (≥40,000)	0.0525	0.0142
BMI ≥25	-0.0083	0.0136
Former smoker	0.0050	0.0120
Current smoker	-0.0015	0.0142
Drinks alcohol	0.0506	0.0124
Exercises	0.1071	0.0104
(Scale)	0.061 [a]	0.0018
(b) GLM - US/RoW cutoffs (reference 0-10)		
(Intercept)	0.5909	0.0466
Mild EDS (ESS, 11-12)	-0.0134	0.0174
Moderate EDS (ESS, 13-15)	-0.0505	0.0165
Severe EDS (ESS, 16-24)	-0.1132	0.0159
OSA without narcolepsy	-0.0238	0.0367
OSA and narcolepsy	-0.1667	0.0626

Age	0.0006	0.0004
CCI	-0.0354	0.0041
Female	-0.0573	0.0119
Married	0.0301	0.0118
Medium Income (20,000-39,999)	0.0498	0.0124
High Income (≥40,000)	0.0538	0.0142
BMI ≥25	-0.0088	0.0136
Former smoker	0.0046	0.0120
Current smoker	-0.0040	0.0142
Drinks alcohol	0.0503	0.0124
Exercises	0.1057	0.0104
(Scale)	0.061 [a]	0.0018
(c) GLM - UK cutoffs (reference 0-10)		
Intercept	0.5918	0.0466
Mild EDS (ESS, 11-14)	-0.0268	0.0138
Moderate EDS (ESS, 15-18)	-0.0789	0.0167
Severe EDS (ESS, 19-24)	-0.1513	0.0236
OSA without narcolepsy	-0.0275	0.0367
OSA and narcolepsy	-0.1640	0.0626
Age	0.0007	0.0004
CCI	-0.0354	0.0041
Female	-0.0575	0.0119
Married	0.0295	0.0117
Medium Income (20,000-39,999)	0.0497	0.0124
High Income (≥40,000)	0.0538	0.0142
BMI ≥25	-0.0107	0.0136
Former smoker	0.0032	0.0120
Current smoker	-0.0048	0.0142

Drinks alcohol	0.0494	0.0124
Exercises	0.1068	0.0104
(Scale)	0.060 [a]	0.0018
(d) Piecewise linear with breakpoint at 11.29		
Intercept	0.6084	0.0480
ESS Slope 1 (ESS, <11.29)	-0.0028	0.0018
ESS Slope 2 (ESS, >11.29)	-0.0134	0.0031
OSA without narcolepsy	-0.0262	0.0368
OSA and narcolepsy	-0.1621	0.0628
Age	0.0006	0.0004
CCI	-0.0353	0.0041
Female	-0.0579	0.0119
Married	0.0295	0.0118
Medium Income (20,000-39,999)	0.0496	0.0124
High Income (≥40,000)	0.0534	0.0143
BMI ≥25	-0.0094	0.0137
Former smoker	0.0045	0.0121
Current smoker	-0.0028	0.0142
Drinks alcohol	0.0495	0.0125
Exercises	0.1061	0.0104
(e) Linear spline with breakpoint at 11		
Intercept	0.6080	0.0479
ESS Slope 1 (ESS, 0-11)	-0.0026	0.0016
ESS Slope 2 (ESS, 12-24)	-0.0131	0.0022
OSA without narcolepsy	-0.0260	0.0368
OSA and narcolepsy	-0.1622	0.0628
Age	0.0006	0.0004
CCI	-0.0352	0.0041

Female	-0.0579	0.0119
Married	0.0295	0.0118
Medium Income (20,000-39,999)	0.0496	0.0124
High Income (≥40,000)	0.0534	0.0143
BMI ≥25	-0.0095	0.0137
Former smoker	0.0045	0.0121
Current smoker	-0.0028	0.0142
Drinks alcohol	0.0496	0.0124
Exercises	0.1060	0.0104

a. Maximum likelihood estimate.

BMI: body mass index; CCI: Charlson Comorbidity Index; EDS: excessive daytime sleepiness; ESS: Epworth Sleepiness Scale; OSA: obstructive sleep apnoea; SE: standard error; RoW: rest of world; UK: United Kingdom; US: United States.

Table S2. Parameter estimates for linear spline regression model: estimation sample

	Estimate	Std. Error	Difference from full sample
Intercept	0.6163	0.0541	0.0083
ESS, 0-11	-0.0019	0.0018	0.0007
ESS, 12-24	-0.0126	0.0025	0.0005
OSA without narcolepsy	-0.0304	0.0415	-0.0043
OSA and narcolepsy	-0.1600	0.0699	0.0022
Age	0.0006	0.0005	<0.0001
CCI	-0.0366	0.0046	-0.0013
Female	-0.0618	0.0134	-0.0039
Married	0.0193	0.0133	-0.0102
Medium Income (20,000-39,999)	0.0506	0.0139	0.0009
High Income (≥40,000)	0.0533	0.0160	-0.0001
BMI ≥25	-0.0102	0.0154	-0.0007
Former smoker	0.0101	0.0135	0.0056
Current smoker	0.0006	0.0158	0.0034
Drinks alcohol	0.0430	0.0139	-0.0066
Exercises	0.1069	0.0117	0.0009

BMI: body mass index; CCI: Charlson Comorbidity Index; EDS: excessive daytime sleepiness; ESS: Epworth Sleepiness Scale; OSA: obstructive sleep apnoea; SE: standard error

Table S3. Fit statistics of estimation sample: overall and by EQ-5D quartile

R2	RMSE	MAE	Prediction Error
0.181	0.244	0.186	0.358
**Quartile**	**RMSE**	**Minimum EQ-5D**	**Maximum EQ-5D**
1st	0.382	-0.358	0.536
2nd	0.100	0.536	0.755
3rd	0.159	0.755	0.887
4th	0.239	0.887	1.000

EQ-5D: EuroQoL- 5 Dimensions; MAE: mean absolute error; RMSE: root mean square error.

Table S4. Slope estimates for piecewise linear regression model with breakpoint of ESS score 11.00: estimation sample

	Estimate	SE	t-value	95% CI
Slope 1 (ESS, 0-11)	-0.0019	0.0021	-0.943	[-0.006; 0.0021]
Slope 2 (ESS, 12-24)	-0.0126	0.0035	-3.603	[-0.0194; -0.0057]

CI: confidence interval; ESS: Epworth Sleepiness Scale; SE: standard error.

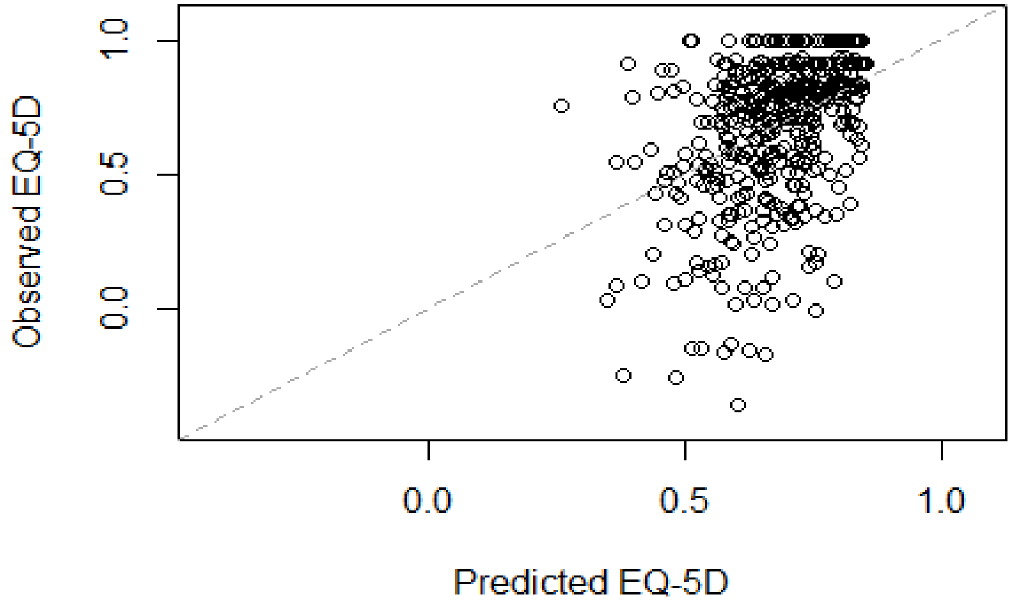

Figure S1. Observed versus predicted EQ-5D utility: estimation sample
EQ-5D: EuroQoL-5 Dimensions.

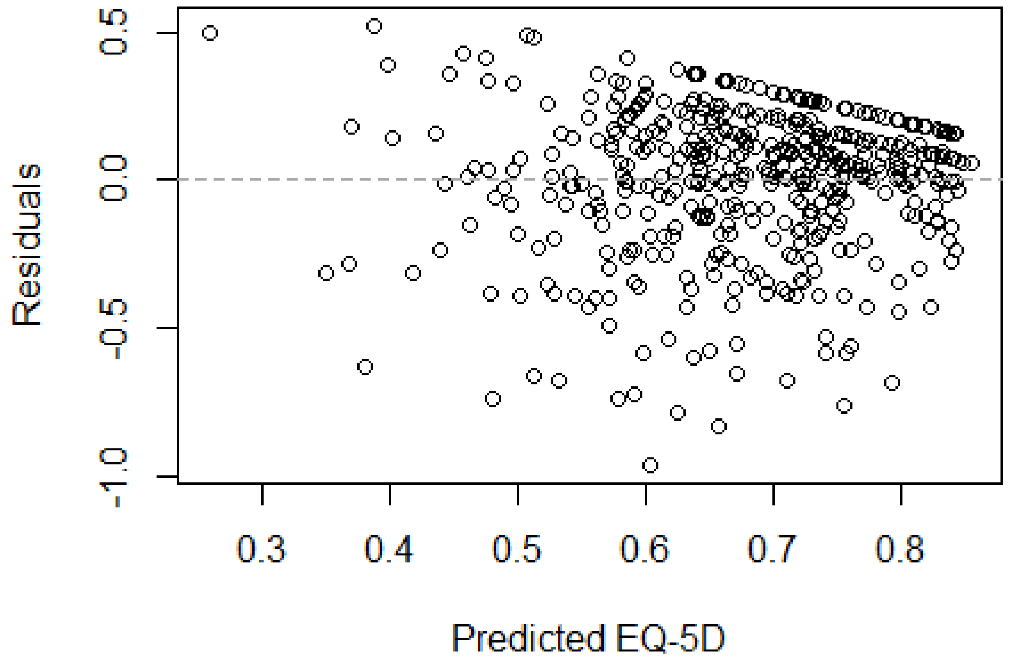

Figure S2. Residual plot for observed versus predicted EQ-5D utility: estimation sample
EQ-5D: EuroQoL-5 Dimensions.
